# Clarifying the Spectrum of Gallbladder Haemorrhage: A Systematic Review of Haemorrhagic Cholecystitis and Haemorrhagic Gallbladder

**DOI:** 10.7759/cureus.94342

**Published:** 2025-10-11

**Authors:** Evangelia Florou, Shirin Ubaid, Carol Balasingh, Parthi Srinivasan, Andreas Prachalias

**Affiliations:** 1 Hepato-Pancreato-Biliary Surgery, King's College Hospital, London, GBR; 2 Hepato-Pancreato-Biliary Surgery and Liver Transplantation, London Bridge Hospital, London, GBR

**Keywords:** gallbladder bleeding, gallbladder rupture, haemobilia, haemorrhagic cholecystitis, haemorrhagic gallbladder, haemorrhagic shock, jaundice

## Abstract

Haemorrhagic cholecystitis (HC) and haemorrhagic gallbladder (HGB) refer to rare clinical entities characterised by bleeding originating from the gallbladder. These terms are often used interchangeably in literature to describe a spectrum of presentations, ranging from incidental findings to life-threatening haemorrhage and shock. Reported cases are scarce and primarily limited to case reports and small series. Clinical presentation and management vary significantly, from conservative antibiotic therapy to urgent surgical intervention. This systematic review aims to summarise the published clinical experience, clarify terminology use, and propose a stratified management approach based on presentation severity.

A systematic review of the literature was conducted in accordance with PRISMA guidelines, covering publications from January 2019 to May 2025. PubMed, Embase, Medline, and Google Scholar were searched using terms including “haemorrhagic cholecystitis”, “haemorrhagic gallbladder”, and “gallbladder haemorrhage”. Only English-language studies reporting extractable patient-level data were included. Data collected included demographics, background comorbidities, anticoagulation status, presenting symptoms, haemodynamic findings, treatment modality, transfusion requirement, and mortality. Cases were stratified as haemorrhagic cholecystitis (HC) or haemorrhagic gallbladder (HGB) based on clinical severity, haemodynamic compromise, and treatment urgency.

A total of 65 cases from 50 publications were included. The median age was 67 years (range 20-92), with a male predominance (63.1%). Anticoagulation was reported in 47.7% of cases. Typical symptoms such as right upper quadrant pain, fever, or vomiting were observed in 90.8%, while jaundice, gastrointestinal bleeding, and haemorrhagic shock occurred in 15.4%, 29.2%, and 16.9% of patients, respectively. Gallstones were present in 38.5% of cases. Multimodal treatment was the most commonly employed approach (30.8%), followed by urgent open cholecystectomy (26.2%) and urgent laparoscopic cholecystectomy (18.5%). The overall mortality rate was 10.8%. Retrospective stratification classified 73.8% of cases as HGB and 26.2% as HC.

HC and HGB represent a continuum of gallbladder haemorrhage, with HC referring to stable patients managed conservatively or electively, and HGB reserved for those with haemodynamic instability requiring urgent intervention. Greater awareness is warranted in elderly patients on anticoagulation and in younger patients with atypical presentations or underlying systemic disease. A stratified management approach based on clinical severity is recommended.

## Introduction and background

Haemorrhagic cholecystitis (HC) and haemorrhagic gallbladder (HGB) are rare but potentially life-threatening conditions involving bleeding within or from the gallbladder. Although often used interchangeably, these terms likely describe different stages of a clinical spectrum, from intraluminal bleeding secondary to haemorrhagic inflammation to more advanced presentations such as gallbladder rupture and hemoperitoneum. Due to their low incidence, most available evidence consists of isolated case reports and small series, limiting the ability to define standardised diagnostic or management pathways [1-3].

Presentations range from non-specific symptoms such as abdominal pain or anaemia to more severe findings, including jaundice, gastrointestinal bleeding, and haemorrhagic shock. Reported management strategies vary accordingly, from conservative therapy to embolisation or urgent surgical intervention [4-6]. In a recent systematic review, Tarazi et al. examined the published literature and emphasised the inconsistent use of terminology and treatment indications. They proposed that classification based on haemodynamic stability and imaging features may help guide appropriate intervention [7].

Building on that concept, this review systematically evaluates the literature from 2019 to 2024 and retrospectively stratifies reported cases into HC or HGB based on clinical parameters such as haemodynamic status, transfusion requirement, haemoglobin drop, and timing of intervention. The objective is to clarify terminology use, assess consistency in reporting, and propose a severity-based management framework for this rare condition.

## Review

Materials and methods

A systematic literature review was conducted in accordance with PRISMA 2020 guidelines. A comprehensive search of PubMed, Embase, Medline, and Google Scholar was performed to identify relevant studies published between January 2019 and May 2025. The search terms included “haemorrhagic cholecystitis”, “haemorrhagic gallbladder”, “gallbladder rupture”, and “haemorrhagic shock and gallbladder rupture”. Only English-language publications reporting clinical cases of gallbladder-origin haemorrhage were included. Eligible studies comprised case reports, case series, and retrospective reviews with extractable patient-level data. Articles were excluded if they lacked clinical detail, described purely histological findings without clinical correlation, or presented aggregated data that could not be stratified. This systematic review was not prospectively registered in a publicly accessible database.

To avoid duplication and maintain relevance to current practice, studies summarised in the pre-2019 systematic review by Tarazi et al. were not re-analysed [7]. Extracted variables included patient demographics, background use of anticoagulants and non-steroidal anti-inflammatory drugs (NSAIDs), presence of gallstones, presenting symptoms, haemoglobin drop, transfusion requirement, treatment modality, and reported mortality.

Treatment strategies were categorised using a 12-point classification system (e.g., conservative management, elective or emergency surgery, interventional radiology, or multimodal approaches). Cases were stratified as haemorrhagic cholecystitis (HC) if patients were haemodynamically stable and managed conservatively, semi-electively, or electively, and as haemorrhagic gallbladder (HGB) if patients were unstable or required urgent or multimodal intervention. Descriptive statistics were used to summarise the findings. Cases with incomplete stratification data were reviewed by two authors and categorised by consensus.

Results

Fifty publications were included in this review, reporting a total of 65 individual cases. Two case series that were excluded need to be mentioned: one due to reliance solely on imaging findings with key clinical variables (e.g., symptoms, haemodynamic status, treatment, and outcomes) inconsistently reported or missing; and the second because cases were identified from histological diagnoses without clinical correlation or documentation of treatment urgency.

Of the 65 patients analysed, 23 were females and 42 males, yielding a female-to-male ratio of approximately 1:1.8. The median age was 67 years, ranging from 20 to 92 years.

Anticoagulation therapy was identified as a potential risk factor in 31 cases (47.7%), while non-steroidal anti-inflammatory drug (NSAID) use was noted in three patients (4.6%). Comorbidities were frequent and often multisystemic. From a cardiovascular perspective, 22 patients (33.8%) had coronary artery disease, atrial fibrillation, or heart failure; cerebrovascular disease, including prior strokes or transient ischaemic attacks, was present in seven cases (10.8%). Renal dysfunction was documented in 13 patients (20.0%), including those with chronic kidney disease and end-stage renal disease. Malignancies were reported in four patients (6.2%), while three patients (4.6%) had a history of solid organ transplantation. Background liver disease, including cirrhosis and portal hypertension, was identified in eight patients (12.3%), and systemic or metabolic conditions such as diabetes, autoimmune disease, or sarcoidosis were seen in 26 cases (40.0%).

Clinical presentation was dominated by typical symptoms such as right upper quadrant pain, vomiting, fever, or a combination of them, reported in 57 cases (87.7%). Jaundice was present in 10 patients (15.4%), while gastrointestinal bleeding occurred in 19 cases (29.2%). Haemorrhagic shock, reflecting the most severe end of the clinical spectrum, developed in 11 patients (16.9%). Notably, many of these patients initially presented with typical or atypical symptoms but progressed to haemodynamic instability during hospitalisation and early treatment. A drop in haemoglobin and/or transfusion requirement was documented in 24 patients (36.9%) and 18 patients (27.7%), respectively, although this information was not consistently reported in all cases.

A wide range of treatments was applied and are summarised in Table 1. Conservative management with antibiotics alone was used in three patients (4.6%), elective cholecystectomy in four cases (6.2%), cystic artery embolisation alone in two (3.1%), and percutaneous cholecystostomy in five cases (7.7%). Percutaneous transhepatic biliary drainage (PTBD) and endoscopic retrograde cholangiopancreatography (ERCP) were used as sole interventions in one case each (1.5%), though they were more often part of a broader multimodal plan. Urgent laparoscopic and urgent open cholecystectomies were performed in 12 (18.5%) and 17 (26.2%) patients, respectively. The most common approach was a multimodal strategy combining interventional radiology or endoscopy with surgery for gallbladder resection, applied in 20 patients (30.8%). Overall, seven patients (10.8%) died, while 58 (89.2%) survived. Gallstones were documented in 25 patients (38.5%), absent in 30 (46.2%), and not reported in 10 cases (15.4%).

**Table 1:** Multimodal treatment application for HC and HGB reported in literature. Treatment modalities applied in 65 cases of HC and HGB. Reported treatments were categorised as conservative (antibiotic therapy alone), elective surgery, urgent surgery (laparoscopic or open cholecystectomy), interventional procedures (cystic artery embolisation, percutaneous drainage), and multimodal approaches (combinations of radiological, surgical, and/or endoscopic interventions). Frequencies and percentages reflect the therapeutic heterogeneity and urgency dictated by clinical severity. *Multimodal treatment includes interventional radiology/endoscopy followed by urgent surgery for gallbladder removal. PTBD: percutaneous transhepatic biliary drainage; ERCP: endoscopic retrograde cholangiopancreatography HC: haemorrhagic cholecystitis; HGB: haemorrhagic gallbladder

Treatment Type	Count	Percentage
Multimodal treatment*	20	30.8
Urgent open cholecystectomy	17	26.2
Urgent laparoscopic cholecystectomy	12	18.5
Cholecystostomy	5	7.7
Elective cholecystectomy	4	6.2
Embolization	2	3.1
Conservative, antibiotics	3	4.6
PTBD	1	1.5
ERCP	1	1.5

HC/HGB stratification

Cases were stratified as haemorrhagic cholecystitis (HC) or haemorrhagic gallbladder (HGB) based on clinical severity, haemodynamic status, and treatment approach. HC was defined by haemodynamic stability and non-urgent treatment, including conservative management, semi-elective or elective cholecystectomy, cholecystostomy, PTBD, or ERCP. These patients typically did not exhibit a haemoglobin drop or require transfusion. In contrast, HGB was characterised by haemodynamic compromise, the presence of shock or gastrointestinal bleeding, a documented drop in haemoglobin or transfusion requirement, and the need for urgent intervention such as embolisation, laparotomy, or a multimodal approach combining surgical and radiological management. Among the 65 analysed cases, 48 (73.8%) were classified as HGB and 17 (26.2%) as HC.

**Figure 1:**
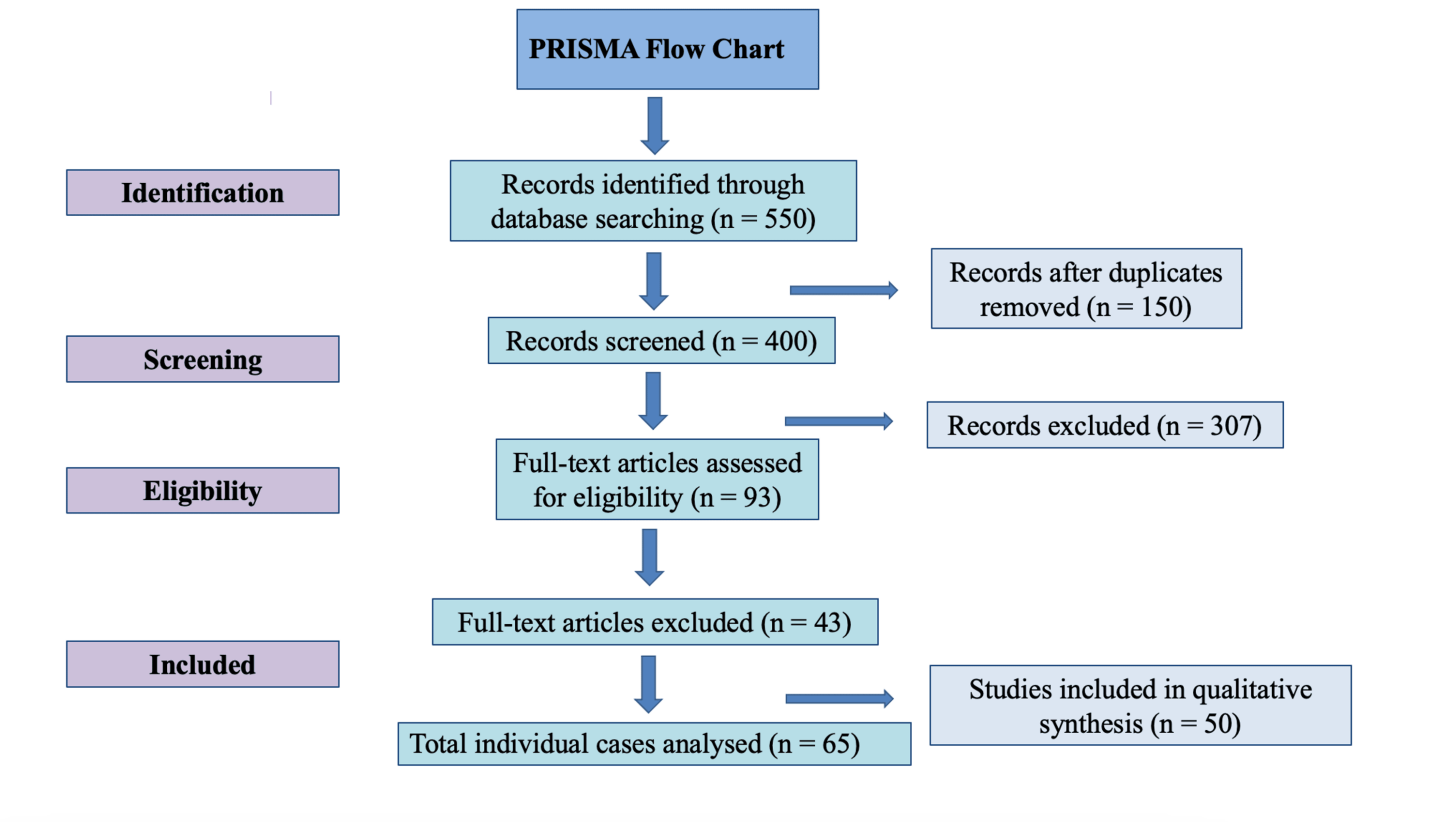
PRISMA flowchart The initial 550 records were retrieved from PubMed, Embase, Medline, and Google Scholar using the terms “haemorrhagic cholecystitis”, “haemorrhagic gallbladder”, “gallbladder rupture”, and “haemorrhagic shock and gallbladder rupture”. After deduplication, 400 records were screened. Forty-three full-text articles were excluded due to lack of extractable clinical data (n = 21), histological-only reports without clinical correlation (n = 14), or duplicate/overlapping cases (n = 8).

In accordance with PRISMA guidelines, a flowchart (Figure 1) and summarised table of all included studies with key clinical and methodological parameters are provided (Table 2). 

**Table 2:** Summary of included studies reporting cases of HC and HGB. Summary of included studies reporting cases of HC and HGB. The table includes author, year of publication, study title, and key clinical parameters: number of cases, demographic data (gender, age), background liver disease, clinical presentation (typical symptoms, jaundice, gastrointestinal bleeding, haemorrhagic shock), treatment modality, transfusion requirement, mortality, and case stratification (HC vs. HGB) based on clinical severity and urgency of intervention. HC: haemorrhagic cholecystitis; HGB: haemorrhagic gallbladder

First author	Year of publication	Title	Number of cases	Gender	Age	Bgx liver disease	Typical symptoms	Jaundice	GI bleed	Haemorrhagic shock	Treatment	Transfusion	Death	HC or HGB
Louis M et al. [8]	2024	Management of NSAID-Induced Penetrating Gastric Ulcer Complicated by Hemorrhagic Cholecystitis: The Role of Percutaneous Transhepatic Biliary Drainage	1	F	68	No	Yes	No	No	No	PTBD	NS	No	HC
AlMoshary M et al. [9]	2024	Hemorrhagic cholecystitis afflicted with glanzmann thrombasthenia patient	1	M	27	No	Yes	Yes	No	No	urgent laparoscopic cholecystectomy	NS	No	HC
Diaz V et al. [10]	2024	Hemorrhagic Cholecystitis Due to Rupture of Underlying Anomalous Duplicated Cystic Arteries	1	M	33	Yes	Yes	No	Yes	No	embolization	Yes	No	HGB
Harrison N et al. [11]	2023	Haemorrhagic cholecystitis in a young patient, complicated by gallbladder perforation and choledocholithiasis	1	F	43	No	Yes	No	Yes	No	conservative, antibiotics	No	No	HC
Omura T et al. [12]	2023	Acute hemorrhagic cholecystitis related to diffuse neurofibroma of gallbladder in a patient with neurofibromatosis type 1	1	M	46	No	Yes	No	No	No	urgent open cholecystectomy	Yes	No	HGB
Morrow J et al. [13]	2023	Haemorrhagic cholecystitis treated with endovascular embolization	1	F	52	No	Yes	No	No	Yes	embolization	Yes	No	HGB
Cochran R et al. [14]	2022	COVID-19 associated spontaneous hemorrhagic cholecystitis	1	M	67	Yes	Yes	No	No	Yes	cholecystostomy	No	No	HGB
Karaisli S et al. [15]	2022	Perforated hemorrhagic cholecystitis in a patient with Bernard–Soulier syndrome	1	M	57	No	Yes	No	No	No	urgent open cholecystectomy	Yes	Yes	HGB
Natcher P et al. [16]	2022	Mirizzi Syndrome in the Setting of Hemorrhagic Cholecystitis	1	M	75	No	Yes	No	No	No	cholecystostomy	NS	No	HC
Aldohayan N et al. [17]	2021	Hemorrhagic cholecystitis with auto-avulsion	1	F	74	No	Yes	No	No	No	urgent open cholecystectomy	NS	No	HGB
Hotak M et al. [18]	2021	A case of haemorrhagic cholecystitis with no risk factors	1	F	51	No	Yes	No	No	No	urgent laparoscopic cholecystectomy	NS	No	HC
Liao TK et al. [19]	2020	Idiopathic Intrahepatic Artery Aneurysm Presenting as Acute Hemorrhagic Cholecystitis	1	F	20	No	Yes	No	No	No	multimodal treatment	No	No	HGB
Long N et al. [20]	2021	Point-of-care ultrasonography in the diagnosis of hemorrhagic cholecystitis	1	M	81	No	Yes	No	No	No	ERCP	NS	No	HC
Rahesh J et al. [21]	2021	Atraumatic spontaneous hemorrhagic cholecystitis	1	F	65	No	Yes	No	No	No	urgent laparoscopic cholecystectomy	NS	No	HGB
Nitta T et al. [22]	2021	Emergency laparoscopic cholecystectomy for hemorrhagic cholecystitis: A case report	1	M	64	No	Yes	No	No	No	urgent laparoscopic cholecystectomy	NS	No	HC
Leaning M et al. [23]	2021	Acalculous hemorrhagic cholecystitis	1	M	73	No	Yes	No	No	No	urgent laparoscopic cholecystectomy	NS	No	HC
Sakharuk I et al. [24]	2021	Anticoagulant-induced hemorrhagic cholecystitis with hemobilia after deceased donor kidney transplant and literature review	1	M	55	No	Yes	No	No	No	urgent open cholecystectomy	NS	No	HGB
Lan X et al. [25]	2019	Massive hemoperitoneum and upper GI hemorrhage following liver rupture secondary to GB perforation	1	M	53	No	Yes	No	Yes	No	urgent open cholecystectomy	NS	No	HGB
Pickell Z et al. [26]	2021	Acute hemorrhagic cholecystitis with gallbladder rupture and massive intra0abdominal hemorrhage	1	M	67	No	Yes	No	Yes	No	urgent open cholecystectomy	Yes	No	HGB
Staszak J et al. [27]	2019	cholecystitis and hemobilia	1	M	43	No	Yes	No	Yes	No	multimodal treatment	NS	No	HGB
Ardu M et al. [28]	2020	Hemoperitoneum from Hemorrhagic Perforated Cholecystitis in a Patient with Acquired Deficiency of Factor VIII	1	M	79	No	Yes	No	No	No	urgent open cholecystectomy	Yes	No	HGB
Zhang X et al. [29]	2020	Hemorrhagic cholecystitis with rare imaging presentation: a case report and a lesson learned from neglected medication history of NSAIDs	1	F	57	No	Yes	No	No	No	elective cholecystectomy	NS	No	HGB
Yam M et al. [30]	2020	A 51-year-old female presenting with shock due to hemorrhagic cholecystitis	1	F	51	No	Yes	No	No	Yes	multimodal treatment	NS	No	HGB
Chen X et al. [31]	2020	A haemorrhagic cholecystitis presenting as obstructive jaundice	1	F	63	No	Yes	Yes	No	No	multimodal treatment	NS	No	HGB
Bergeron E et al. [32]	2019	Massively distended, necrotic and hemorrhagic gallbladder in a long-term octreotide-treated patient with added everolimus	1	F	63	No	Yes	Yes	No	No	multimodal treatment	NS	No	HGB
Donn et al. [33]	2019	Hemorrhagic Cholecystitis after Warfarin Use for Deep Vein Thrombosis	1	M	63	No	Yes	No	No	No	multimodal treatment	Yes	No	HGB
Ng Z et al. [34]	2019	Haemorrhagic cholecystitis: a rare entity not to be forgotten	1	F	68	No	Yes	No	No	Yes	urgent open cholecystectomy	NS	No	HGB
Lauria A et al. [35]	2019	Hemorrhagic Cholecystitis: An Uncommon Disease Resulting in Hemorrhagic Shock	1	M	73	Yes	Yes	No	Yes	No	urgent open cholecystectomy	Yes	Yes	HGB
Jiang et al. [36]	2020	Hemorrhagic Cholecystitis	1	M	68	No	Yes	No	No	Yes	urgent open cholecystectomy	Yes	Yes	HGB
Cirillo B et al. [37]	2020	Acalculous hemorrhagic cholecystitis and SARS - CoV-2 infection	1	M	79	No	Yes	No	No	No	urgent laparoscopic cholecystectomy	NS	No	HGB
Eduardo S et al. [38]	2024	Hemorrhagic cholecystitis. A challenge for the general surgeon	1	M	35	No	Yes	No	No	No	urgent open cholecystectomy	NS	No	HGB
Gobishangar S et al. [39]	2021	Hemorrhagic cholecystitis: A rare cause of melena	1	F	75	No	Yes	Yes	Yes	No	elective cholecystectomy	Yes	No	HC
Anouassi Z et al. [40]	2023	A case of haemorrhagic cholecystitis in a patient on apixaban after covid 19 infection	1	M	67	No	Yes	Yes	No	No	conservative , abx	Yes	No	HGB
Arscott T et al. [41]	2024	Hemorrhagic cholecystitis: A case report	1	M	69	No	Yes	No	No	No	urgent laparoscopic cholecystectomy	NS	No	HC
Khoury G et al. [42]	2019	An intraoperatively diagnosed case of hemorrhagic cholecystitis in a 43 year old patient: case report	1	F	43	No	Yes	No	No	No	urgent laparoscopic cholecystectomy	NS	No	HC
Hasegawa T et al. [43]	2021	A case of hemorrhagic cholecystitis and hemobilia under anticoagulation therapy	1	M	70	No	Yes	Yes	No	No	multimodal treatment	NS	No	HGB
Torrico CP et al. [44]	2024	Severe hemorrhagic cholecystitis in the absence of common predisposing factors	1	M	70	No	Yes	No	No	No	urgent open cholecystectomy	NS	No	HGB
Espejo N et al. [45]	2024	Hemorrhagic Cholecystitis: the forgotten differential diagnosis	1	M	59	No	Yes	No	Yes	No	multimodal treatment	Yes	No	HGB
Tarazi M et al. [7]	2019	literature review and case series of haemorrhagic cholecystitis 1	1	M	87	No	Yes	No	No	No	cholecystostomy	NS	No	HC
Tarazi M et al. [7]	2019	literature review and case series of haemorrhagic cholecystitis 2	1	F	65	No	Yes	No	No	No	conservative , abx	NS	No	HC
Tarazi M et al. [7]	2019	literature review and case series of haemorrhagic cholecystitis 3	1	F	92	No	Yes	No	No	No	cholecystostomy	NS	No	HC
Baier A et al.[46]	2022	Emergent laparoscopic surgical intervention for perforated hemorrhagic cholecystitis with hemodynamic instability 1	1	M	74	No	Yes	No	No	No	urgent laparoscopic cholecystectomy	Yes	No	HGB
Baier A et al. [46]	2022	Emergent laparoscopic surgical intervention for perforated hemorrhagic cholecystitis with hemodynamic instability 2	1	M	69	No	Yes	No	Yes	No	urgent laparoscopic cholecystectomy	Yes	No	HGB
Baier A et al. [46]	2022	Emergent laparoscopic surgical intervention for perforated hemorrhagic cholecystitis with hemodynamic instability 3	1	F	23	Yes	Yes	No	No	Yes	urgent laparoscopic cholecystectomy	Yes	No	HGB
Chernopolsky PM et al. [47]	2024	Hemorrhagic cholecystitis - two cases and literature review 1	1	F	68	No	Yes	Yes	No	No	urgent open cholecystectomy	NS	No	HGB
Chernopolsky PM et al. [47]	2024	Hemorrhagic cholecystitis - two cases and literature review 2	1	M	76	No	Yes	No	No	No	urgent open cholecystectomy	NS	No	HGB
Kim HC et al. [48]	2023	patient 1 out of 10 Transcatheter arterial embolization of cystic artery bleeding	1	M	43	Yes	No	No	No	No	multimodal treatment	NS	No	HGB
Kim HC et al. [48]	2023	patient 2 out of 10 Transcatheter arterial embolization of cystic artery bleeding	1	F	49	Yes	No	No	Yes	No	multimodal treatment	NS	Yes	HGB
Kim HC et al. [48]	2023	patient 3 out of 10 Transcatheter arterial embolization of cystic artery bleeding	1	M	75	No	No	No	Yes	No	multimodal treatment	NS	No	HGB
Kim HC et al. [48]	2023	patient 4 out of 10 Transcatheter arterial embolization of cystic artery bleeding	1	M	74	No	Yes	No	Yes	No	multimodal treatment	NS	Yes	HGB
Kim HC et al. [48]	2023	patient 5 out of 10 Transcatheter arterial embolization of cystic artery bleeding	1	M	48	No	Yes	No	Yes	No	multimodal treatment	NS	Yes	HGB
Kim HC et al. [48]	2023	patient 6 out of 10 Transcatheter arterial embolization of cystic artery bleeding	1	F	78	No	Yes	No	Yes	No	multimodal treatment	NS	No	HGB
Kim HC et al. [48]	2023	patient 7 out of 10 Transcatheter arterial embolization of cystic artery bleeding	1	M	78	No	Yes	No	No	No	multimodal treatment	NS	No	HGB
Kim HC et al. [48]	2023	patient 8 out of 10 Transcatheter arterial embolization of cystic artery bleeding	1	M	64	No	Yes	No	Yes	No	multimodal treatment	NS	No	HGB
Kim HC et al. [48]	2023	patient 9 out of 10 Transcatheter arterial embolization of cystic artery bleeding	1	M	80	Yes	No	No	Yes	No	multimodal treatment	NS	Yes	HGB
Kim HC et al. [48]	2023	patient 10 out of 10 Transcatheter arterial embolization of cystic artery bleeding	1	M	80	No	No	No	Yes	No	multimodal treatment	NS	No	HGB
Florou E et al. [49]	2025	hemorrhagic GB on BGx of PSC	1	F	29	Yes	Yes	Yes	No	Yes	urgent open cholecystectomy	Yes	No	HGB
Sweeney A et al. [50]	2019	Hemorrhagic cholecystitis causing hemobilia and common bile duct obstruction	1	M	78	No	Yes	Yes	No	No	elective cholecystectomy	No	No	HC
Reens D et al. [51]	2019	Hemorrhagic cholecystitis: A case of expecdited diagnosis by point -of -care ultrasound in the emergency department	1	M	76	No	Yes	No	No	No	cholecystostomy	NS	No	HC
Shah R et al. [52]	2020	Hemorrhagic Cholecystitis in a Patient with Cirrhosis and Rectal Cancer	1	M	66	Yes	Yes	No	Yes	Yes	urgent laparoscopic cholecystectomy	Yes	No	HGB
Nguyen D et al. [53]	2021	Acute Hemorrhagic Cholecystitis with Large Hemoperitoneum: Treatment with Microcoil Embolization and Subsequent Cholecystectomy 1	1	M	74	No	No	Yes	No	Yes	multimodal treatment	NS	No	HGB
Nguyen D et al. [53]	2021	Acute Hemorrhagic Cholecystitis with Large Hemoperitoneum: Treatment with Microcoil Embolization and Subsequent Cholecystectomy 2	1	M	74	No	No	No	No	Yes	multimodal treatment	Yes	No	HGB
Ma Z et al. [54]	2019	Anticoagulants is a risk factor for spontaneous rupture and hemorrhage of gallbladder: a case report and literature review	1	F	51	No	Yes	No	No	Yes	urgent open cholecystectomy	NS	No	HGB
Itagaki H et al. [55]	2019	Gallbladder hemorrhage during orally administered edoxaban therapy: a case report	1	F	86	No	No	No	Yes	No	elective cholecystectomy	NS	No	HC
Azam M et al. [56]	2021	It's the Bloody Gallbladder! Spontaneous Gallbladder Hemorrhage Following Factor Xa Inhibition	1	M	55	No	Yes	No	No	No	urgent open cholecystectomy	NS	No	HGB

Discussion

HC and HGB are terms often used interchangeably to describe the rare clinical scenario of bleeding originating from the gallbladder. The condition is known to represent a rare complication in the context of cholecystitis; however, it has been primarily documented through isolated case reports and small series, contributing to the lack of standardised diagnostic and management protocols [1,2,49].

In their literature review, Tarazi et al. analysed 31 cases reported between 1985 and 2018, identifying anticoagulation therapy as the most common risk factor, present in 45% of patients. Interestingly, an equal proportion (45%) were not receiving anticoagulation, and in 10% of cases, anticoagulation status was not reported [7]. These findings partially align with those of the present review, in which anticoagulation was identified in 47.7% of cases. This substantial proportion highlights a strong association, though not necessarily causation [7,33]. A retrospective study further demonstrated that although anticoagulated patients undergoing cholecystectomy experienced increased intraoperative blood loss and transfusion requirements, their postoperative outcomes were not significantly worse compared to non-anticoagulated counterparts [57]. These findings collectively support the interpretation that anticoagulation likely functions as a risk-enhancing cofactor in haemorrhagic gallbladder pathology, particularly when mucosal integrity is compromised, a fact that can be encountered in both acute and chronic cholecystitis settings.

Other potential contributors identified included chronic renal failure, haemophilia, vasculitic disorders, steroid use, and advanced age. While Tarazi et al. highlighted the potential contribution of systemic illness, the broader dataset in this review reinforces the multifactorial nature of haemorrhagic gallbladder pathology and its strong association with comorbidities [7]. Notably, 47.7% of patients who developed HGB had more than one underlying systemic illness. These findings underscore the need for risk-based stratification and heightened clinical vigilance, particularly in vulnerable patient populations.

While classic symptoms such as right upper quadrant pain, vomiting, and fever were observed in 89.2% of cases, less typical features like jaundice (15.4%) and gastrointestinal bleeding (29.2%) were also reported. These atypical signs may appear concurrently or sequentially, potentially delaying diagnosis.

Jaundice is likely a result of haemobilia obstructing the biliary tract, and if unrecognised, gastrointestinal bleeding may become the next evident clinical manifestation. On the contrary, diagnosis is more challenging when patients present with GI bleeding and, in the absence of common symptoms, work-ups fail to direct clinicians to identify that the cause of bleeding is haemobilia [58].

Haemobilia refers to bleeding into the biliary tree, typically resulting from vascular erosion, trauma, inflammation, or tumour invasion. When blood enters the common bile duct, it can obstruct biliary flow, resulting in biochemical evidence of cholestasis. This manifests as an obstructive pattern on liver function tests, including elevated bilirubin, alkaline phosphatase, and gamma-glutamyl transferase levels. Clinically, this may present as jaundice, often in the absence of gallstones or malignancy [59,60].

Gallstones were documented in 38.5% of cases, while 46.2% had no evidence of cholelithiasis, and 15.4% lacked documentation. Notably, gallstones were present across both HC and HGB categories, without a clear trend correlating their presence to severity. This suggests that gallstones, while common, are not essential for the development of haemorrhagic manifestations. When stratified by clinical severity, gallstones were found in 10 out of 15 HC cases (66.7%), but in only 15 of 50 HGB cases (30%). This suggests that gallstones may be more commonly associated with stable, less severe presentations of gallbladder haemorrhage, whereas HGB is more often observed in the absence of gallstones. These findings imply that gallstone-related inflammation might trigger haemorrhage in HC, while HGB likely reflects alternative mechanisms such as chronic cholecystitis, mucosal ischaemia, vascular compromise, or anticoagulation-related bleeding.

The above findings reflect the pathophysiological mechanisms of acute cholecystitis known so far. Acute cholecystitis begins with cystic duct obstruction, most commonly due to gallstones, which leads to bile stasis and elevated intraluminal pressure in the gallbladder [61]. This increased pressure causes venous congestion and mucosal oedema, reducing blood flow and precipitating mucosal ischaemia and necrosis [61,62]. Histologically, the gallbladder wall shows inflammation, oedema, vascular congestion, haemorrhage, neutrophilic infiltration, and focal mucosal necrosis [62]. Continued ischaemia may progress to gangrene and perforation, sometimes accompanied by bacterial invasion from organisms, which exacerbates transmural inflammation [63]. In acalculous cholecystitis, particularly in critically ill patients, hypoperfusion rather than stone obstruction is the primary driver of ischaemic injury, leading to similar histopathological findings [62,63]. Overall, acute cholecystitis can occur in both settings, with or without gallstones.

In the context of haemorrhagic cholecystitis, transmural inflammation with or without vascular compromise of the gallbladder wall can lead to intraluminal bleeding that extends into the cystic duct and biliary tree, manifesting as obstructive jaundice [61,65-66]. The pathophysiological mechanisms underlying this process, particularly in the setting of recent or ongoing anticoagulation or in the presence of systemic disease, remain poorly understood. However, literature suggests that intracystic bleeding may be triggered by ischaemia due to increased intraluminal pressure and compromised perfusion, possibly exacerbated by anticoagulants, trauma, or systemic illness [31,36,52]. Histopathologically, the gallbladder wall demonstrates oedema, vascular congestion, subserosal haemorrhage, neutrophilic infiltration, mucosal necrosis, and full-thickness necrosis or infarction have also been documented [17,67]. A small subset of patients presenting with such atypical features rapidly progressed to haemorrhagic shock, representing the most severe end of the clinical spectrum [52]. These findings, along with the occurrence of haemodynamic compromise, haemoglobin drop, or transfusion requirement in a substantial proportion of cases, support once again the rationale for stratifying presentations into haemorrhagic cholecystitis (HC) and HGB.

In our review, treatment approaches were notably heterogeneous, reflecting both the clinical severity of presentation and the anatomical complexity encountered. Tarazi et al. reported cholecystectomy as the predominant treatment modality, performed in 71% of the 31 cases reviewed, with only a small proportion managed conservatively or by cholecystostomy [7]. In contrast, our review identified a broader therapeutic landscape. While urgent cholecystectomy (laparoscopic or open) was undertaken in 44% of cases, the most frequently reported intervention was a multimodal approach, combining interventional radiology, endoscopy, and/or surgery, used in 30.5% of patients. Additionally, less common but significant interventions such as cystic artery embolisation (3.4%), elective cholecystectomy (3.4%), and percutaneous techniques like PTC (1.7%) or ERCP (1.7%) highlight the nuanced, case-specific nature of treatment. Conservative management with antibiotics alone was rare (5.1%) and limited to haemodynamically stable individuals.

These findings show the variability in management approaches, which were primarily guided by the patient’s clinical status.

The differences in treatment trends between studies likely reflect the absence of a symptom-based stratification model to guide management and show inconsistencies in the reporting and documentation of haemorrhagic cholecystitis across the literature, potentially contributing to an underestimation of its true severity.

Stratification may enhance diagnostic suspicion when common symptoms suggestive of gallbladder pathology are accompanied by more serious or unusual clinical features. It can also help guide the urgency and nature of clinical interventions, accounting for patient-specific factors such as age, baseline functional status, and comorbidities.

Supportive of our suggestion of the need for a stratified approach to haemorrhagic gallbladder pathology is the large retrospective study by Hotak et al., which reviewed 35 cases of histologically confirmed haemorrhagic cholecystitis identified from over 6400 cholecystectomy specimens [68]. Notably, only 5.7% of patients were on anticoagulation therapy, and nearly 89% underwent laparoscopic cholecystectomy, indicating a stable preoperative clinical status in the majority. While their cohort adds epidemiological weight to the incidence of HC (0.55%), it provides limited insight into clinical urgency, haemodynamic compromise, or symptom severity, as the diagnosis was made histologically post-resection. Furthermore, the inclusion of predominantly elective or emergency cholecystectomy specimens, with all patients surviving without complication, reinforces the view that not all haemorrhagic findings carry acute clinical significance. This highlights the value of a clinical classification into HC and HGB, particularly in guiding decision-making when haemorrhage is suspected preoperatively, based on atypical presentations or physiological instability. This study was excluded from the final cohort of studies analysed in our review due to its reliance on histopathological diagnosis alone, with a lack of detailed clinical presentation, haemodynamic status, treatment urgency, or outcome data, thus the essential parameters required for stratification and meaningful clinical comparison.

Similarly, the retrospective case series by Calderón et al. reported 11 patients with radiologically confirmed haemorrhagic cholecystitis based on ultrasound and computed tomography (CT) imaging [69]. While this study offers valuable insight into imaging characteristics, such as haemobilia (91%), hemoperitoneum (55%), and ductal bleeding manifestations, it was excluded from our analysis. Crucially, it lacked comprehensive clinical data on patient presentation, comorbidities, haemodynamic status, timing or urgency of intervention, treatment modalities, and patient outcomes. These omissions precluded meaningful comparison and stratification in our analysis.

In conclusion, both the Hotak and Calderón series offer contrasting perspectives on the clinical significance, severity, treatment application, and reported incidence of bleeding from the gallbladder, thus collectively highlighting the urgent need for standardised case documentation. Their divergence reinforces the importance of adopting a structured stratification framework based on clinical presentation, haemodynamic stability, and treatment urgency to enable more accurate reporting and a deeper, more consistent understanding of HC and HGB [68,69].

This review proposes a pragmatic stratification model based on clinical severity, with HC referring to haemodynamically stable patients managed conservatively or with elective intervention, and HGB representing unstable cases requiring urgent and/or multimodal treatment. Applying this model retrospectively, 48 of 65 cases (73.8%) met criteria for HGB, characterised by urgent intervention and haemorrhagic shock or a documented haemoglobin drop and blood transfusion. This underscores the life-threatening nature of HGB in the majority of patients. Although the overall mortality rate was 10.8%, stratification likely provides a more meaningful reflection of disease burden, with deaths predominating in the HGB subgroup. These findings reinforce the importance of standardised terminology, documentation, and a severity-based management approach tailored to patient physiology and clinical context.

## Conclusions

HC and HGB represent a rare but clinically significant spectrum of gallbladder pathology, often presenting with overlapping symptoms and variable haemodynamic consequences. This systematic review highlights the heterogeneity in clinical presentation, risk factors, and treatment approaches, and reveals critical gaps in standardised terminology and reporting.

By introducing a stratification model based on clinical severity, distinguishing stable HC from unstable HGB, we propose a practical framework to enhance diagnostic suspicion, guide the urgency of intervention, and support consistent documentation. Wider adoption of this classification may improve recognition, allow for more accurate incidence reporting, and ultimately optimise patient outcomes through tailored management strategies.
